# Are biosimilars the next tool to guarantee cost-containment for pharmaceutical expenditures?

**DOI:** 10.1007/s10198-013-0538-4

**Published:** 2013-11-23

**Authors:** María-Isabel Farfan-Portet, Sophie Gerkens, Isabelle Lepage-Nefkens, Irmgard Vinck, Frank Hulstaert

**Affiliations:** Belgian Health Care Knowledge Centre, Boulevard du Botanique, 50, 1000 Brussels, Belgium

## Introduction

Biological medicines contain a biological substance that is produced by or derived from a living organism. The active substances of biologicals are usually larger and more complex than those of chemically derived medicines (non-biological medicine). Biologicals are used for the treatment of chronic and life-threatening diseases such as cancer, multiple sclerosis and rheumatoid arthritis. Treatment with biologicals is usually expensive and represents ever-increasing pharmaceutical expenditures for the third-party payer.

In analogy with the introduction of generics for chemically derived medicines, the expiration of patents of the first biological medicines opened new hopes for affordable copies and increased competition. The replicate versions of a biological medicine “the so-called biosimilars” are available on the European market since 2006, 2007 and 2008, for growth hormone, erythropoiesis-stimulating agents and granulocyte-colony stimulating factors, respectively. In June 2013, the Committee for Medicinal Products for Human Use (CHMP) recommended granting marketing authorisations for the first two monoclonal antibody biosimilars (infliximab).

Most of the available literature on biosimilars has focused on the critical analysis of their specific market authorization procedure. Before 2010, almost no literature addressed theoretical or empirical questions on biosimilar competition and its subsequent impact on uptake and price erosion. This editorial looks at these issues and provides an overview on economic aspects of the new biosimilar competition.

## Can biosimilar competition resemble generic competition?

A brief reminder of the differences between generics and biosimilars is required before presenting evidence on biosimilar competition. Whilst generics are considered to be exact copies of chemically derived medicines, biosimilars are considered not identical but rather similar to the originator medicine [1]. According to the European Medicines Agency (EMA) approved biosimilars and each reference medicinal product are expected to have the same safety and efficacy profile and are generally used to treat the same conditions [1]. The EMA developed a pioneer pathway for biosimilar market authorisation, which includes specific guidelines on how to address similarity for different biological medicines. Requirements of the biosimilar pathway do not include all elements of a complete dossier for the approval of a new medicine but are more stringent than the requirements for the approval of generics. Owing to these scaled down market authorisation requirements for biosimilars, it is expected that pharmaceutical companies can produce biosimilars at a lower cost while ensuring their quality, safety and efficacy.

Biosimilar competition 1will most probably differ from generic competition. First, it is widely accepted that production costs for biological medicines are higher than for chemically derived molecules. In principle, this also holds for differences in the production costs of biosimilars and of generics [2–8]. A possible consequence of these higher costs is that ultimately fewer firms will produce biosimilars. Today, only one biosimilar is commercialized (Sandoz) in the growth hormone product class. For the erythropoiesis-stimulating agents and granulocyte colony-stimulating factors product classes, five and six biosimilars are being commercialized, respectively. However, only two different manufacturers (Rentschler Biotechnologie and Norbitec) produce the five epoetin biosimilars. The same is true for the filgrastim biosimilars for which three manufactures share the production of the six commercialized products [9, 10]. Another determinant of the overall cost of a medicine is the information service offered by the marketing authorisation holder. The level of service for the medical community associated with generics and biosimilars is reportedly low as compared with the originator product. However, in order to familiarize physicians with the concept of biosimilarity, high investments in information services could still be needed for biosimilar medicines.

Second, the International Nonproprietary Name (INN) 2cannot be used, as for generics [12], to reduce market failure arising from identification of medicines for biosimilars and the originator biological medicine. INNs for biological medicines are more problematic than for chemically derived medicines because of the lack of a homogenous chemical structure [13]. INN prescribing is usually not allowed for biological medicines in Europe [14] and it is a common practice that the logging of a physician prescription for biological medicines includes the lot number and the manufacturers name (brand name) to ensure their traceability and distinguishability (Article 102 of the medicinal products Directive 2001/83/EU, as amended by Directive 2010/84/EU) [15]. In addition, there is a debate within the medical and pharmaceutical community on whether biosimilars need a distinct INN from their originator and/or from each other. The European innovative biotechnology and pharmaceutical industry associations plead in favour of a distinct INN for biosimilars [16] whereas the European Generics Association (EGA) argues that comparability of two biologicals is sufficient to assign the same INN [17]. For currently available biosimilars in Europe, the naming situation is rather complex and even confusing. For instance, three biosimilars for the epoetin class share the same INN “epoetin alfa” of the originator Eprex^®^ and two have their unique INN “epoetin zeta”. Biosimilars and originator medicines share the same INN for filgrastim class and for the growth hormone (the INN “filgrastim” of Neupogen^®^ and the INN “somatropin” of Humanotrope^®^). 3

Different theoretical frameworks have been used in two articles to analyse biosimilar competition [6, 7]. Grabowski et al. [7] use a monopolistic competition model to explain how large investment costs relating to biosimilar production lead to fewer competitors and less price erosion than in markets facing generic competition. In their paper, Grabowski et al. expect that the price difference between a biosimilar and an originator medicine will attain 10, 25 and 67 % after market entrance of one, three or twelve biosimilars, respectively. Chauhan et al. [6] adapt Frank and Salkever’s [18] generic competition model to reflect biosimilars competition. Chauhan et al. [6, 7] use a duopolistic market model (biosimilar and originator) where there is a degree of product differentiation between the biosimilar and the originator medicine. Competition between the originator and the biosimilar depends on the price-sensitive and non-price-sensitive portion of the market. Given that there is product differentiation, the originator medicine benefits from a monopolistic position in the non-price-sensitive “loyal” market segment. On the contrary, competition between the biosimilar and the originator medicine exists in the price-sensitive “non-loyal” section of the market. Compared with price erosion created by generic competition, the two models predict a lower difference between the price of the biosimilar and of the originator product. However, Chauhan et al. expect that larger experience with biosimilars will enhance competition and may result in further price erosion.

## Have biosimilars led to price erosion?

Current evidence on price differences between the biosimilar and the originator medicine is limited, probably because biosimilar competition is a recent phenomenon. Broadly, price difference between the originator medicine and the biosimilars ranged between 10 and 35 % (see Table 1). Rovira et al. [2] use the mean price per daily defined dosage (DDD) across 24 European countries to calculate the price differences between the biosimilar and the originator product. Other studies also provide information per country [14, 19], for one molecule and for different countries [20] or a global estimation [5]. Two studies reported country-specific information from national authorities [2, 14].

**Table 1:** Price difference between the originator product and the biosimilars

	Year	Country	Source	Product specific			Non-product specific (%)
				Epoetin (%)	Somatropin (%)	Filgrastim (%)	
Moran [5]^a^	NA	NA	Reported by pharmaceutical companies	NA	NA	NA	10–35
Hughes [19]	2009	UK	British National Formulary (BNF)	10–25			
Rovira et al. [2]^b^	2009	Average 24 European countries	IMS data	17	14.1	35.0	
	2009	Italy	National authorities	15	20	22	
	2009	Spain					30
Lepage-Nefkens et al. [14]	2012	Belgium	National authorities	30–34	22	20	
Liefner [20]	2007	Germany	Simon-Kucher and partners	30			
	NA	Finland			0		
	NA	Denmark			5		
	NA	Spain			19		
	NA	UK			21		
	NA	France			21		
	NA	Germany			25		

*NA* not available,* IMS* Intercontinental Marketing Services

^a^Product- or country-specific information was not mentioned

^b^Rovira et al. also reported country estimates based on IMS data; for detailed country-specific estimates please refer to their work

## How much biosimilar uptake?

**Fig. 1:**
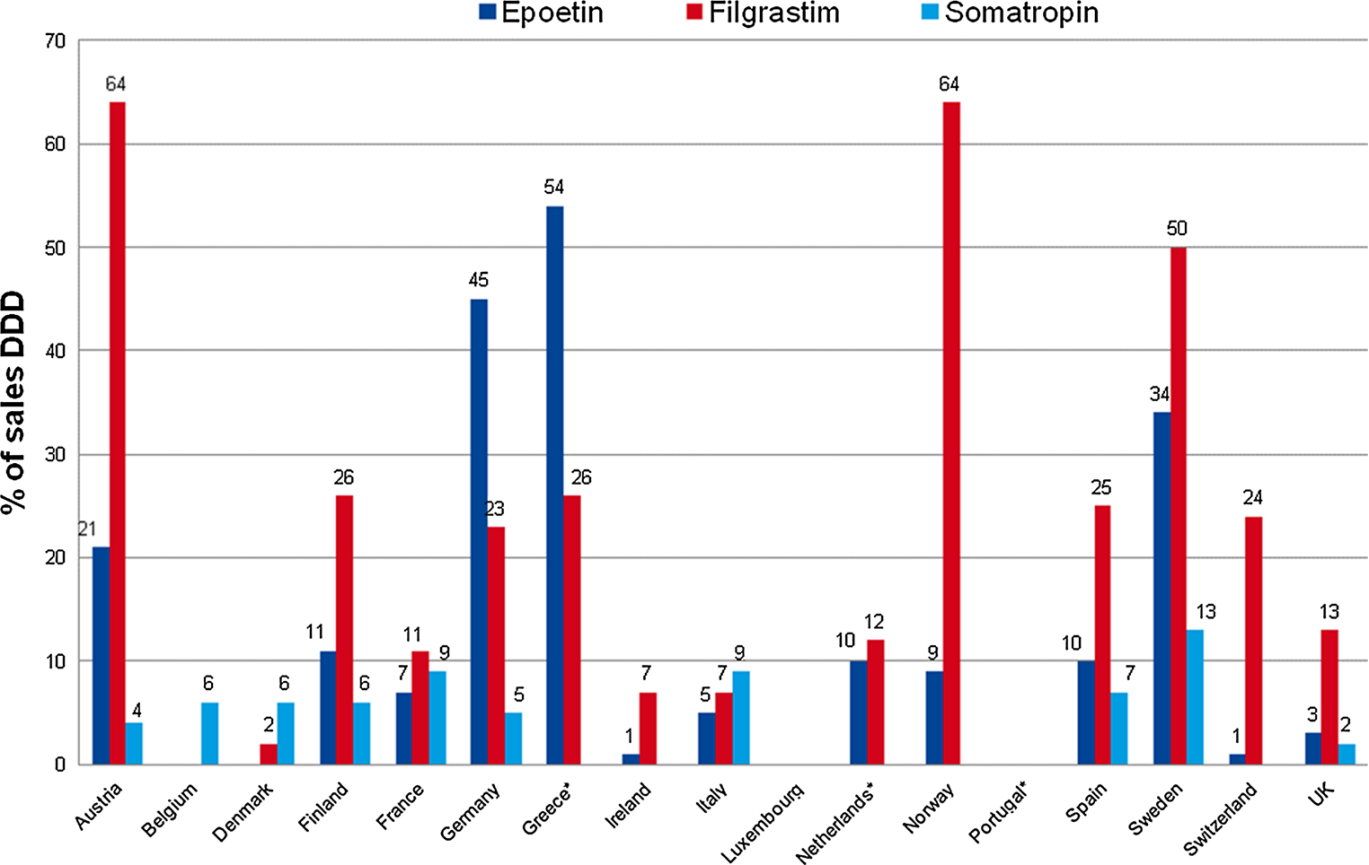
Percentage of sales in DDD of biosimilars of total market (biosimilars, reference product and non-reference product). *Source* IMS data 2nd trimester 2011 [23]. *Only retail sector.* DDD* defined daily dose. Second-generation products not included

Most information on biosimilar uptake is based on the data provided by IMS Health [2, 4, 21, 22]. Figure 1 provides the most recent available information on biosimilar uptake in a sample of European countries [23]. Market shares for biosimilars are calculated as a percentage of DDD in each product class. Product classes include biosimilars and originator products as well as me-too pharmaceuticals (second-generation products are excluded). Biosimilar sales (in DDDs) are still a relatively small segment of the EU pharmaceutical market, but have strong annual growth [23]. Market shares of filgrastim biosimilars are highest in Austria, Norway and Sweden. Uptake for epoetin biosimilars was highest in Germany, Greece and Sweden. The uptake of somatropin biosimilar is generally lower than for the other two product classes (filgrastim and epoetin). This may be related to the fact that somatropin is used for growth-hormone-related illnesses which require long-term treatment whereas medicines containing epoetin and filgrastim are used for short-term treatment. The highest uptake for somatropin biosimilars was found in Sweden, France and Italy. Belgium, Luxembourg and Portugal are lingering behind, with no uptake or with uptake limited to only in one product class (somatropin).

## What savings can be attributed to the introduction of biosimilars?

Rovira et al. [2] mentioned that comparability of estimates on biosimilar-related savings is limited as the data are based on different modelling strategies and are dependent on the hypothesis used by different analysts. In addition, estimates for Europe mostly come from the pharmaceutical sector itself and include savings relating to groups of medicines for which biosimilars are not yet available on the market (e.g. monoclonal antibodies). Consequently, there is a lack of independent analyses of the current and future savings from the use of biosimilars. Several authors reported estimates from the EGA where savings could attain up to €1.6 billion conditional on a 20 % price reduction for five patent-expired biologicals [3, 5, 8, 22]. This rough estimate does not provide information based on the currently available biosimilars but ratter based on expectation of their apparition. Moreover, savings are based on total expenditures neglecting price sensitivity and its impact on purchased volumes.

A recent study used IMS data to provide estimates on biosimilar-related savings between 2007 and 2020 for eight European countries (Germany, France, the UK, Italy, Spain, Sweden, Poland and Romania) [24]. Expected savings are calculated for epoetins and for filgrastim for which biosimilars are already commercialized. Scenarios for possible savings relating to the future apparition of biosimilars in the class of monoclonal antibodies were also included. Expected savings for the eight countries and between 2007 and 2020 vary from €11.8 billions (slow penetration and minimal price reduction) to €33.4 billion (fast penetration and maximal price reduction) [24]. While these findings provide the most recent estimates on biosimilar-related savings, they rely on heavy assumptions concerning outcomes of the German generic pharmaceutical market (extrapolated to other countries) and to large savings obtained within the monoclonal antibodies class (mAbs) for which biosimilars are not yet available on the market. For many countries, the hypothesis used by the authors can overestimate biosimilar-related savings as (1) the Germany generic market is more developed than that of other countries (and therefore overestimating uptake) and (2) for complex molecules (such as mAbs) it is not know whether biosimilars will enter the market immediately after that the originator patent expires [25].

## Discussion points for the future of biosimilar competition

As for generics the biggest advantage of biosimilars is that they may offer a less-expensive alternative to an existing medicine and therefore reduce pharmaceutical expenditure for the third-party payer. However, regulatory issues, biosimilar acceptability among physicians, price and reimbursement policies as well as supply and demand-side incentives will ultimately determine the actual level of biosimilar-related savings.

Theoretical models predicted that biosimilar competition will lead to less price erosion than that obtained through generic competition [6, 7]. In line with this theoretical prediction, although price erosion arising from generic competition of up to 80 % has been reported in countries like the UK and Germany, reported price erosion from biosimilar competition has not exceeded 35 % [2, 5, 14, 19, 20].

The hypothesis of segmentation for biosimilar markets is also supported by real-life evidence and may depend on physician loyalty (and lack of tools to circumvent it), perception of product differentiation and patient type (new patients and patients already following a treatment). Without pharmacist substitution or the possibility to prescribe by INN and if physicians are sceptical to switch a patient from an originator medicine to a biosimilar, market uptake for biosimilars can only take place among new users. Even with regard to treatment-naïve patients, physicians facing different information or incentives may overlook the financial advantages of prescribing biosimilars. For instance, because biological medicines are usually prescribed for life-threatening or chronic illness, patient cost-sharing is usually limited [14]. Therefore, prescription decisions based on prices or cost will depend only on physician attitudes regarding insurer cost (or third-party payer). Physicians may also be more reluctant to use biosimilars for certain molecules or populations. For instance, despite strong competition between originator products in the growth hormone class (somatropin), it is a common practice to maintain patients on the same treatment. Somatropin is mostly prescribed to children and a learning process related to the companion medicine device is an important part of the compliance process [14]. Some authors reported that one barrier limiting the uptake of the somatropin biosimilar resided on the difference between the device of the biosimilar and of the originator medicine [4, 14].

Gaining shares in the price-sensitive part of the market as well as reducing (at least to some extent) market segmentation will critically depend on experience with biosimilars that will in return influence perception of product differentiation. This may require transmitting to healthcare professionals clinical data proving the effectiveness and safety of biosimilars, including data obtained after switching. Moreover, appropriate investment in commercial and marketing strategies will be needed to make prescribers aware of the possibilities and qualities of these less expensive alternatives. Compared with generics, a different marketing approach may be needed as biosimilars are considered as molecules that are more complex and because policy measures to circumvent physician loyalty are currently limited. These elements seem to highlight that biosimilar competition may resemble more that of me-too pharmaceuticals than that of generic medicines [4, 6, 7].

Expiry of market exclusivity of major biological blockbusters is the main driver surrounding the interest in the development of the biosimilar industry. Many leading “traditional” originator companies are already developing biosimilars. Companies’ experience in the production of complex biologicals may lead to optimized production of biosimilars at low cost and even drive originators to reconsider their production method. Originator companies will probably produce biosimilars in new product classes (for instance mAb) and may have different marketing strategies towards health professionals than current biosimilar manufacturers. Whether these companies will use the same strategies and provide similar levels of information services for their innovator products and for biosimilars remains an open question. Yet, this may change the current perception of biosimilars and even the current biosimilar business model.

The challenge for policy makers in the coming years will be to set effective measures leading to improved biosimilar uptake. Policy makers need to envisage that policy measures that have been successful in increasing generic use, such as INN prescribing, may not be currently appropriate to promote biosimilar uptake. Expectations on future savings related to forthcoming biosimilars are a key driver for interest and concern from national authorities on biosimilar current market penetration. Lack of market penetration of the currently available biosimilars may be seen as a lost opportunity, less in terms of current savings than as a barrier for potential future savings. In line with this, more evidence needs to be provided on the impact of public policies in stimulating biosimilar uptake.

## Appendix

A structured review of the literature was performed up to 8 November 2012 in MedLine OVID and PUBMED with the following terms: “biosimilar*” or “biosimilar pharmaceuticals/MeSH”. A complementary search was done in institutional and grey literature databases (DRIVER, OAISTER and Google Scholar). Only articles containing empirical data, theoretical models as well as discussions or reviews were included. From 735 initially identified references, 35 articles and one book remained. Full texts were then searched and inclusion/exclusion criteria were applied on full texts. Abstract were only available for nine articles [26–34]; three articles [35–37] could not be accessed. Seven articles did not comply with the inclusion criteria [38–44]. In addition five articles discussing cost-effectiveness of biosimilars [45–49] were not included because evidence on efficacy and safety was not analysed as a part of this review. A total of 11 articles and one book were considered relevant and were included in our review. We also included information from the project group Market Access and Uptake of Biosimilars [23] and from one recent report on biosimilar uptake in Belgium [14].

## Footnotes

1 A structured literature review was performed. Details can be found in the “Appendix” section.

2 The International Nonproprietary Name (INN) is a unique name that is globally recognised and is public property (also referred to as “generic name”). In principle, the INN is selected only for a single, well-defined substance that can be unequivocally characterized by a chemical name (or formula) [11].

3 For the purpose of clarity, in the rest of the text we refer to erythropoiesis-stimulating agents as epoetin, granulocyte colony-stimulating factors as filgrastim and growth hormone as somatropin.

## References

[1] Directive 2001/83/EC of the European Parliament and of the Council of 6 November 2001 on the Community code relating to medicinal products for human use. Off. J. Eur. Union 28 November 2001, L 311

[2] Rovira J, Espín J, García L, Olry de Labry A (2011). The Impact of Biosimilars’ Entry in the EU Market.

[3] Borget I, Grivel T (2010). Biosimilaires et facteurs medico-economiques. Bull. Cancer.

[4] Grabowski H, Long G, Mortimer R (2011). Implementation of the biosimilar pathway: economic and policy issues. Seton Hall Law Rev..

[5] Moran N (2008). Fractured European market undermines biosimilar launches. Nat. Biotechnol..

[6] Chauhan D, Towse A, Mestre Ferrandiz J (2009). The Market for Biosimilars: Evolution and Policy Options.

[7] Grabowski HG, Ridley DB, Schulman KA (2007). Entry and competition in generic biologics. Manag. Decis. Econ..

[8] Ruiz S, Calvo G (2011). Similar biological medicinal products: lessons learned and challenges ahead. J. Generic Med..

[9] Minghetti P, Rocco P, Del Vecchio L, Locatelli F (2011). Biosimilars and regulatory authorities. Nephron.

[10] European Medicines Agency (EMA): European public assessment reports. http://www.ema.europa.eu/ema/index.jsp?curl=pages%2Fmedicines%2Flanding%2Fepar_search.jsp&murl=menus%2Fmedicines%2Fmedicines.jsp&mid=WC0b01ac058001d124&searchTab=searchByAuthType&alreadyLoaded=true&isNewQuery=true&status=Authorised&status=Withdrawn&status=Suspended&status=Refused&keyword=Enter+keywords&searchType=name&taxonomyPath=&treeNumber=&searchGenericType=biosimilars&genericsKeywordSearch=Submit (2012). Accessed 1 Feb 2013

[11] World Health Organisation (1997). Guidelines on the Use of International Nonpropietary Names (INNs) for Pharmaceutical Substances.

[12] Feldman, R., Lobo, F.: Competition in prescription drug markets: the roles of trademarks, advertising, and generic names. Eur. J. Health Econ. **14**, 667–675 (2013) 10.1007/s10198-012-0414-7 22815099

[13] Rader RA (2011). Nomenclature of new biosimilars will be highly controversial. BioProcess Int..

[14] Lepage-Nefkens, I., Gerkens, S., Vinck, I., Piérart, J., Hulstaert, F., Farfán-Portet, M.-I.: Barriers and opportunities for the uptake of biosimilar medicines in Belgium. In: Health Services Research (HSR), vol. 199. Belgian Health Care Knowledge Centre (KCE), Brussels (2013)

[15] Directive 2010/84/EU of the European Parliament and of the Council of 15 December 2010 amending, as regards pharmacovigilance, Directive 2001/83/EC on the Community code relating to medicinal products for human use. Off. J. Eur. Union L 348/74

[16] Chantelot E (2006). Introduction—Naming of Biotechnology Medicines.

[17] European Generic Medicines Association (EGA): EGA position paper on naming of biopharmaceuticals: a contribution to WHO “Review of International Nonproprietary Names (INN) for Biological and Biotechnological Substances”. EGA, Brussels (2006)

[18] Frank RG, Salkever DS (1997). Generic entry and the pricing of pharmaceuticals. J. Econ. Market. Strategy.

[19] Hughes DA (2010). Biosimilars: evidential standards for health technology assessment. Clin. Pharmacol. Ther..

[20] Liefner M, Matisson N, Mestre-Ferrandiz J, Towse A (2009). Biosimilars: price dynamics in Europe. Biosimilars: How Much Entry and Price Competition Will Result?.

[21] Sheppard A (2011). Navigating the biosimilars market: the market landscape for biosimilars is in flux, with limited penetration now, but with the potential for growth for those who can navigate the market. BioPharm Int..

[22] Cornes P (2012). The economic pressures for biosimilar drug use in cancer medicine. Target. Oncol..

[23] Project Group on Market Access and Uptake of Biosimilars: What you need to know about biosimilar medicinal products. A consensus information document. European Commission, Brussels (2013)

[24] Haustein R., de Millas C., Höer, A., Häussler, H.: Saving money in the European healthcare systems with biosimilars. GaBi J.** 1**(3–4), 6 (2011)

[25] Miletich J, Eich G, Grampp G, Mounho B (2011). Biosimilars 2.0: guiding principles for a global “patients first” standard. MAbs.

[26] Hurtado P, Vieta A, Espinos B, Badia X (2011). Market access barriers for biosimilars in Spain and Germany: Epoetin ALFA example. Value Health.

[27] Kani C, Litsa P, Alexopoulou E, Hatzikou M, Delibasi S, Geitona M (2011). Striving for affordable treatments within the Greek environment: Do epoetin biosimilars help?. Value Health.

[28] Lebboroni M, Rosti G, Cerchiari A, Katz P (2012). Health economics analysis of pegfilgrastim in the prophylaxis of febrile neutropenia (FN) in Italy. Eur. J. Hosp. Pharm. Sci. Pract..

[29] Lindner L, Gimenez E, Rovira J, Espin J, Olry A, Leticia G (2011). Biosimilars in the European market. Value Health.

[30] Loiacono C, Sgroi C, Coppolino S, Cannata A, Ferrara R, Arcoraci V, Cananzi P, Savica V, Schuemie M, Caputi AP, Trifiro G (2012). How much are biosimilars used in southern Italy?: a retrospective analysis of epoetin utilization in the local health unit of Messina in the years 2010–2011. BioDrugs.

[31] Long M, Trout J, Akpinar P (2009). Biosimilars: HGH to TNFs, how will payers respond?. Value Health.

[32] Shepelev J, Rauland M, Krattiger C (2011). Biosimilars are not generics from payer perspective. Value Health.

[33] Toscani M, Vogenberg R, Nash D, Peskin S (2011). Issues associated with biologic agents: healthcare stakeholder survey. Value Health.

[34] Viejo Viejo I (2011). Does launch price actually matter?. Value Health.

[35] Blackstone EA, Fuhr JP (2010). Biosimilars and innovation: an analysis of the possibility of increased competition in biopharmaceuticals. Future Med. Chem..

[36] Howell, P.R.: Patient access issues and opportunities. Contract Pharma. http://www.contractpharma.com/issues/2012-05/view_features/access-to-biosimilars/ (2012). Accessed 1 Feb 2013

[37] Jommi C (2010). Biosimilar drugs: Economic issues and Italian market perspectives. Pharmacoecon. Italian Res. Articles.

[38] Aapro MS (2011). What does a prescriber think of biosimilars?. Oncologie.

[39] Doloresco F, Fominaya C, Schumock GT, Vermeulen LC, Matusiak L, Hunkler RJ, Shah ND, Hoffman JM (2011). Projecting future drug expenditures: 2011. Am. J. Health Syst. Pharm..

[40] Grabowski HG, Kyle M, Mortimer R, Long G, Kirson N (2011). Evolving brand-name and generic drug competition may warrant a revision of the Hatch-Waxman Act. Health Aff..

[41] Heinzl S (2008). Epoetin biosimilar: cost-saving potential is still not exhausted. Pharmazeutische Zeitung.

[42] Hoffman JM, Li E, Doloresco F, Matusiak L, Hunkler RJ, Shah ND, Vermeulen LC, Schumock GT (2012). Projecting future drug expenditures–2012. Am. J. Health Syst. Pharm..

[43] Howell P, Burich M (2012). Will biosimilars fulfill their promise of affordability?. Pharm. Process..

[44] Mellstedt H (2009). Biosimilar products-cheaper version of biological drugs: new possibilities within several therapeutic fields. Läkartidningen.

[45] Aapro M, Cornes P, Sun D, Abraham I (2012). Comparative cost efficiency across the European G5 countries of originators and a biosimilar erythropoiesis-stimulating agent to manage chemotherapy-induced anemia in patients with cancer. Ther. Adv. Med. Oncol..

[46] Simoens S (2009). Health economics of market access for biopharmaceuticals and biosimilars. J. Med. Econ..

[47] Simoens S (2011). Biosimilar medicines and cost-effectiveness. Clinicoecon. Outcomes Res..

[48] Simoens S, Verbeken G, Huys I (2011). Market access of biosimilars: not only a cost issue. Oncologie.

[49] Simoens, S., Verbeken, G., Huys, I.: Biosimilars and market access: a question of comparability and costs? Target. Oncol. **7**(4), 227–231 (2012) 10.1007/s11523-011-0192-7 22249657

